# Association Between Wnt Target Genes and Cortical Volumes in Alzheimer’s Disease

**DOI:** 10.1007/s12031-023-02122-1

**Published:** 2023-12-23

**Authors:** Liling Dong, Bo Hou, Caiyan Liu, Chenhui Mao, Xinying Huang, Li Shang, Shanshan Chu, Bin Peng, Liying Cui, Feng Feng, Jing Gao

**Affiliations:** 1 Neurology Department, State Key Laboratory of Complex Severe and Rare Diseases, Peking Union Medical College Hospital, Chinese Academy of Medical Sciences and Peking Union Medical College, Shuaifuyuan No. 1, Dongcheng District, Beijing, 100005 China; 2 Radiology Department, State Key Laboratory of Complex Severe and Rare Diseases, Peking Union Medical College Hospital, Chinese Academy of Medical Sciences and Peking Union Medical College, Shuaifuyuan No. 1, Dongcheng District, Beijing, 100005 China

**Keywords:** Alzheimer’s disease, Wnt signaling pathway, Cortical volume, Cortical atrophy, MRI

## Abstract

The disproportionate cortical atrophy is an established biomarker for the pathophysiological process of Alzheimer’s disease (AD). However, the genetic basis underlying the cortical atrophy remains poorly defined. Herein, we aim to illustrate the effect of the Wnt target genes on the cortical volumes of AD patients. 82 sporadic AD patients were recruited. All the subjects had history survey, blood biochemical examination, cognitive assessment, MRI morphometry and whole exome sequencing. This report focused on 84 common variants (minor allele frequency > 0.01) of 32 Wnt target genes, including the *APC*, *DAAM1*, *DACT1*, *DISC1*, *LATS2*, *TLR2*, *WDR61*, and the *AXIN*, *DVL*, *FZD*, *LRP*, *TCF/LEF*, *WNT* family genes. The Wnt target genes showed asymmetric effects on the cortical volumes of AD patients. The right temporal/parietal/occipital cortices were more affected than left temporal/parietal/occipital cortices. Nevertheless, the reverse applied to the frontal cortex. The *DACT1* affected the cortical thickness most, followed by the *TCF3* and *APC*. The *DACT1* rs698025-GG genotype displayed greater right temporal pole and left medial orbito-frontal gyrus than rs698025-GA genotype (2.4 ± 0.4 vs. 2.0 ± 0.6, P = 0.005; 5.2 ± 0.6 vs. 5.0 ± 0.6, P = 0.001). The brain region most influenced by the Wnt target genes was the right calcarine cortex. In conclusion, the common variants of the Wnt target genes exert asymmetric effects on the cortical volumes of AD patients. The Wnt signaling pathway may play a role in the cortical atrophy of AD patients.

**Supplementary Information:**

The online version contains supplementary material available at 10.1007/s12031-023-02122-1.

## Introduction

Alzheimer's disease (AD) is pathologically characterized by amyloid-beta (Aβ) accumulation and phosphorylated tau deposition. The disproportionate cortical atrophy on structural magnetic resonance imaging (MRI) is an established biomarker for the pathophysiological process of AD (McKhann et al. 2011). The cortical atrophy typically occurs early in the medial temporal lobe and extends along a temporal–parietal–frontal trajectory (Pini et al. 2016). However, the genetic architecture underlying the cortical atrophy remains poorly defined.

The *APOE*-ɛ4 is the strongest genetic risk factor for AD. With a systematic review and meta-analysis of 14 cross-sectional studies, Liu et al., concluded that the *APOE*-ɛ4 is primarily relevant to hippocampal volume (Liu et al. 2015). With the data from Alzheimer’s Disease Neuroimaging Initiative and Allen Human Brain Atlas, Acosta et al., suggested that the *APOE*-ɛ4 did not contribute to the regional atrophy of AD (Acosta et al. 2018). Anyway, more than half of AD patients do not harbor the *APOE*-ɛ4 (Ward et al. 2012). Therefore, more research is required to explore the genetic basis of cortical atrophy in AD.

In recent years, the Wnt signaling pathway is supposed to play a role in AD pathogenesis. It can prevent the amyloidogenic processing of APP by inhibiting the *BACE1* transcription. It can suppress the tau phosphorylation by decreasing the GSK3β activity, which is a kinase related to tau phosphorylation. Besides, the Wnt signaling pathway facilitates neuronal survival and neurogenesis, promotes synaptic plasticity, modulates microglial activity and neuroinflammation, enhances the integrity and function of blood–brain barrier (Inestrosa and Varela-Nallar 2014; Jia et al. 2019). Therefore, the inhibition of Wnt signaling pathway can lead to Aβ42 accumulation, tau phosphorylation, and eventually the occurrence of AD.

In 2020, Grasby et al., deployed a genome-wide association study (GWAS) of brain imaging data from 51,665 individuals. They found that the genetic loci affecting the cortical structures clustered near the genes which were related to the Wnt signaling pathway, such as the *DACT1*, *DAAM1* and *WNT2B* (Grasby et al. 2020).

We hypothesize that the Wnt target genes exert effects on the cortical thickness of AD patients. In this study, firstly, we will identify the Wnt target genes with literature review, Gene Expression Omnibus (GEO) and Uniprot database analysis. Secondly, we will investigate the effect of Wnt target genes on the cortical volumes in AD patients. Ultimately, we expect to better understand the role of Wnt signaling pathway in AD.

## Method

### Participants and Inclusion Criteria

82 unrelated participants were recruited. The inclusion criteria were as follows: 1. Intact data on history survey, blood biochemical examination, cognitive assessment, brain MRI morphometry and whole exome sequencing. 2. Right handedness. 3. Diagnosed with probable AD based on the 2011 diagnostic criteria from National Institute on Aging and the Alzheimer’s Association (McKhann et al. 2011). 4. Sporadic cases with no dementia-causing variants were detected. 5. Written informed consent was acquired.

### Gene Sequencing and Wnt Target Genes

The Genomic DNA were obtained from the peripheral blood. The fragmented DNA were captured by the Agilent SureSelect Human All ExonV6 Kit (Agilent Technologies, Santa Clara, CA, USA) and sequenced on the Illumina Novaseq 6000 platform (Illumina Inc., San Diego, CA, USA).

This report focused on the common variants of the Wnt target genes. The common variants met the following criteria: 1. Minor allele frequency > 0.01 in 1000genomes, GnomAD, ESP6500, ExAC dababases; 2. Located in the exons or adjacent introns (within 10 bp of splicing junction).

The identification of the Wnt target genes were performed with literature review, GEO and Uniprot database analysis. Three GEO datasets were downloaded, including GSE132903, GSE33000 and GSE63061 (https://www.ncbi.nlm.nih.gov/gds/). The differentially expressed genes (DEGs) between AD and control groups were achieved by GEO2R online tool, with adjusted P < 0.01 as the cutoff value. The overlapping DEGs from the three datasets were identified by Venn diagram. According to the uniprot database (https://www.uniprot.org), the overlapping DEGs involved in the Wnt signaling pathway were included in the study.

### MRI Morphometry and Target Cortical Regions

MRI scan was performed on the 3.0 Tesla scanner (Discovery MR750, GE) with an 8-channel head coil. The structural images were acquired by sagittal T1-weighted three-dimensional fast spoiled gradient echo sequence (repetition time = 8.2 ms, echo time = 3.2 ms, flip angle = 12°, Prep Time = 400 ms, matrix = 256 × 256). Morphometry analysis was performed by SPM 12 techniques. According to the tutorial of voxel-based morphometry, the original images were segmented into gray matter, white matter and cerebrospinal fluid. The segmentation images were normalized to Montreal Neurological Institute space. The normalized gray matter images were smoothed with an 8mm^3^ full width at half maximum filter.

This report focused on 56 cortical regions, including 22 temporal (bilateral hippocampus, parahippocampus, entorhinal cortex, insula, fusiform, superior/middle/inferior/transverse temporal gyrus, bank of superior temporal sulcus, temporal pole), 16 frontal (bilateral superior frontal gyrus, anterior/posterior middle frontal gyrus, frontal pole, orbital gyrus, medial/lateral orbitofrontal gyrus, precentral gyrus), 10 parietal (bilateral postcentral, supramarginal gyrus, superior/inferior parietal lobule, precuneus), and 8 occipital areas (bilateral cuneus, lingual, calcarine, lateral occipital cortex).

### Statistics

As for each single nucleotide polymorphism (SNP), the MRI morphometry data were compared by analysis of covariate and post hoc Bonferroni correction. Age, gender, disease course, *APOE* status (ε4 carriers or non-carriers) and whole brain volume were included in the model as fixed factors or covariates.

## Result

### Demographic Characteristics

As shown in Table 1, 36.6% (30/82) participants were males and 63.4% (52/82) were females. The average age was 73.6 ± 9.6 years old. The average disease course was 3.4 ± 2.4 years. The *APOE*-ε4 allele frequency in the cohort was 0.26. 58.5% (48/82) cases were *APOE*-ε4 non-carriers, 31.7% (26/82) were ε4 heterozygotes, and 9.8% (8/82) were ε4 homozygotes. All the participants were right handedness.

**Table 1:** Demographic data of 82 participants

Gender (Male/Female) (n (%))	30 (36.6) / 52 (63.4)
Age (years old)	73.6 ± 9.6
Disease course (years)	3.4 ± 2.4
*APOE*-ε4 (+ + / + -/–) (n (%))	8 (9.8) / 26 (31.7) / 48 (58.5)
ε2ε2 (n (%))	0 (0)
ε2ε3 (n (%))	8 (9.8)
ε3ε3 (n (%))	40 (48.8)
ε2ε4 (n (%))	1 (1.2)
ε3ε4 (n (%))	25 (30.5)
ε4ε4 (n (%))	8 (9.8)

*APOE* apolipoprotein e

### Wnt Target Genes and SNPs

Literature review indicated that several genes were involved in the Wnt signaling pathway, including the *APC*, *CTNNB1*, *DAAM1*, *DACT1*, *GSK3B*, *LRP5-6*, as well as the *AXIN*, *DVL*, *FZD*, *TCF/LEF* and *WNT* family genes (Cheyette et al. 2002; Jia et al. 2019; Li et al. 2005; Liu et al. 2008; Tapia-Rojas et al. 2016; Zhang et al. 2006).

**Fig. 1:**
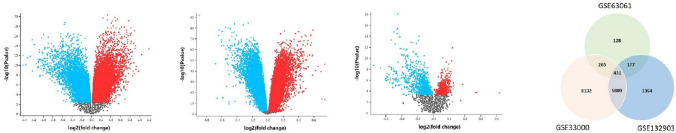
Volcano plots and Venn diagram of GSE132903, GSE33000, GSE63061. The first three volcano plots were from GSE132903, GSE33000, GSE63061, respectively. The differentially expressed genes between AD and control groups were achieved by GEO2R online tool, with adjusted P < 0.01 as the cutoff value. The fourth was the Venn diagram for the overlapping differentially expressed genes of the three datasets

The GSE132903, GSE33000 and GSE63061 were RNA expression profiles from middle temporal gyrus, prefrontal cortex and peripheral blood, respectively. There were 9762 DEGs between AD and control groups in GSE132903, 14,628 DEGs in GSE33000, and 1001 DEGs in GSE63061. A total of 431 overlapping DEGs from the three datasets were identified (Fig. 1; Supplement Table S1). According to the uniprot database, six genes were implicated in the Wnt signaling pathway, including the *CSNK1G2*, *DISC1*, *LATS2*, *PFDN5*, *TLR2* and *WDR61* (https://www.uniprot.org).

This report focused on the common variants of the above Wnt target genes. Some genes were excluded since no common variants were available in the cohort, such as the *CTNNB1*, *GSK3B*, *CSNK1G2*, *PFDN5*, etc. Finally, 84 SNPs of 32 Wnt target genes were included in this study. The 32 Wnt genes included the *APC*, *AXIN1*, *AXIN2*, *DAAM1*, *DACT1*, *DISC1*, *DVL2*, *FZD1*, *FZD3*, *FZD6*, *FZD8*, *FZD10*, *LATS2*, *LEF1*, *LRP5*, *LRP6*, *TCF3*, *TCF4*, *TCF7*, *TCF7L2*, *TCF12*, *TLR2*, *WDR61*, *WNT2B*, *WNT7A*, *WNT8A*, *WNT8B*, *WNT9A*, *WNT9B*, *WNT10B*, *WNT11* and *WNT16*. The 84 Wnt target SNPs were shown in Table 2.

**Table 2:** 84 Wnt target SNPs and alternate allele frequencies in the cohort

Gene	SNP	AAF		Gene	SNP	AAF		Gene	SNP	AAF		Gene	SNP	AAF
*APC*	rs2229992	0.70		*DISC1*	rs3738401	0.23		*LATS2*	rs558614	0.57		*TCF4*	rs8766	0.48
	rs351771	0.82			rs3738402	0.27			rs7317471	0.88		*TCF7*	rs30489	0.41
	rs41115	0.82			rs2492367	0.11			rs77919685	0.03		*TCF12*	rs35615435	0.30
	rs42427	0.82			rs12133766	0.05		*LEF1*	rs4956157	0.92		*TCF7L2*	rs77961654	0.27
	rs866006	0.82			rs821616	0.17		*LRP5*	rs314776	0.10		*TLR2*	rs3804099	0.32
	rs459552	0.90			rs11122391	0.05			rs4988319	0.09			rs3804100	0.28
	rs465899	0.82			rs821617	0.17			rs545382	0.98		*WDR61*	rs3832993	0.12
*AXIN1*	rs214252	0.09			rs2273890	0.08			rs2277268	0.08			rs2280364	0.09
	rs214250	0.09		*DVL2*	rs35594616	0.50			rs2306862	0.18		*WNT10B*	rs1051886	0.58
	rs1805105	0.34			rs222837	0.65			rs4988322	0.08		*WNT11*	rs1533767	0.21
*AXIN2*	rs35415678	0.31			rs222836	0.50			rs556442	0.71		*WNT16*	rs2908004	0.18
	rs1133683	0.32			rs2074216	0.38			rs3736228	0.19			rs2707466	0.18
	rs9915936	0.80		*FZD1*	rs139480179	0.27		*LRP6*	rs2302685	0.89		*WNT2B*	rs910697	0.60
	rs2240307	0.18		*FZD10*	rs10848026	0.54		*TCF3*	rs55677929	0.70		*WNT7A*	rs3762719	0.52
	rs2240308	0.31		*FZD3*	rs2241802	0.56			rs62130064	0.05			rs12639607	0.52
*DAAM1*	rs8022614	0.62		*FZD6*	rs3736047	0.57			rs2074888	0.27		*WNT8A*	rs6596422	0.65
	rs941884	0.82			rs3808554	0.57			rs1140828	0.17		*WNT8B*	rs3793771	0.15
*DACT1*	rs17832998	0.18			rs3808553	0.57			rs8140	0.76		*WNT9A*	rs8192629	0.08
	rs2003021	0.32			rs1053917	0.56			rs1052696	0.10			rs3795768	0.59
	rs698025	0.15		*FZD8*	rs74989785	0.15			rs1052692	0.09		*WNT9B*	rs4968281	0.45
	rs863091	0.15		*LATS2*	rs59928188	0.04			rs2240590	0.21			rs34072914	0.02

*SNP* single nucleotide polymorphism, *AAF* alternate allele frequency in the cohort

### Wnt SNP Frequency

Table 2 demonstrated the alternate allele frequencies of the 84 SNPs in the cohort. Compared with the East Asian population from GnomAD database, the AD patients in this study showed lower allele frequencies in three *TCF3* SNPs, including rs1052692 (0.09 vs. 0.23), rs1052696 (0.10 vs. 0.23) and rs2074888 (0.27 vs.0.41). The allele frequencies of the remaining 81 SNPs in this AD group were close to those of the East Asian population (Table 2; Supplement Table S2).

### Effect of Wnt SNPs on MRI Morphometry

**Fig. 2:**
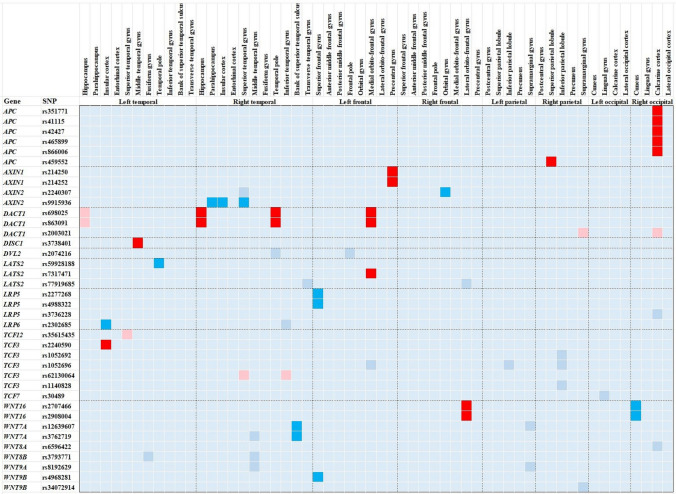
Effect of Wnt target SNPs on cortical volumes of AD patients. This illustrated the effect of 38 Wnt target SNPs on 56 cortical regions of AD patients. Each square represents the specific effect of the SNP of the horizontal axis on the cortex of the vertical axis. The different colors of these squares represent different P-values. P < 0.05; P < 0.01, the subjects with the alternate alleles show smaller cortical volumes than those with reference alleles. P < 0.05; P < 0.01, the subjects with the alternate alleles show greater cortical volumes than those with reference alleles. P ≥ 0.05. The remaining 46 Wnt SNPs with no effects on cortical thickness were not shown in this figure

Among the 84 Wnt target SNPs, 45.2% (38/84) SNPs showed effects on cortical volumes (Fig. 2; Supplement Table S3). The raw values were shown below (mm^3^).

Among the *APC* and *AXIN* SNPs, the 351,771-A allele was related to smaller right calcarine cortex (GG vs. GA, AA: 2.7 ± 0.7 vs. 2.3 ± 0.5, 2.1 ± 0.4, P = 0.004). So were the alternate alleles of rs41115, rs42427, rs866006 and rs465899. The rs459552-TA genotype showed greater right superior parietal lobule than rs459552-AA (11.4 ± 1.4 vs. 10.6 ± 1.5, P = 0.004). The rs9915936-G allele was relevant to greater right parahippocampus, insula and superior temporal gyrus (AA vs. AG, GG: 2.7 ± 0.5 vs. 3.4 ± 0.7, 3.4 ± 0.6, P = 0.001; 4.8 ± 0.6 vs. 6.1 ± 0.8, 6.0 ± 0.8, P < 0.001; 8.1 ± 2.4 vs. 10.1 ± 1.4, 10.2 ± 1.3, P = 0.009). The rs2240307-C allele was associated with greater right orbital gyrus (TT vs. TC, CC: 2.4 ± 0.4 vs. 2.6 ± 0.4, 2.7 ± 0.4, P = 0.001).

Among the *DACT1*, *DISC1* and *LAST2* SNPs, the rs698025-GG genotype displayed greater right temporal pole and left medial orbito-frontal gyrus than rs698025-GA genotype (2.4 ± 0.4 vs. 2.0 ± 0.6, P = 0.005; 5.2 ± 0.6 vs. 5.0 ± 0.6, P = 0.001). So was the rs863091-CC genotype relative to rs863091-CT. Also, the rs7317471-AG genotype exhibited greater left medial orbito-frontal gyrus relative to rs7317471-GG (5.4 ± 0.6 vs. 5.1 ± 0.6, P = 0.005). In addition, the rs3738401-G allele was associated with greater left middle temporal gyrus (GG, GA vs. AA: 9.4 ± 1.5, 9.3 ± 1.5 vs. 7.1 ± 1.9, P = 0.005). The rs59928188-GG genotype showed smaller left temporal pole than rs59928188-GA (2.4 ± 0.3 vs. 2.7 ± 0.4, P = 0.005).

Among the *LRP* and *TCF* SNPs, the rs2277268-GG phenotype showed smaller left superior frontal gyrus than rs2277268-GA (19.0 ± 2.4 vs. 20.4 ± 2.9, P = 0.005). So was the rs4988322-TT genotype relative to rs4988322-TC. The rs2302685-GA and rs2240590-CT genotypes exhibited smaller left insula than rs2302685-AA and rs2240590-CC, respectively (5.5 ± 0.7 vs. 5.9 ± 0.8, P = 0.004; 5.5 ± 0.7 vs. 5.9 ± 0.7, P = 0.003).

Among the *WNT* SNPs, the rs2908004-G allele correlated with greater right lateral orbitofrontal gyrus (GG, GA vs. AA: 6.5 ± 0.9, 6.3 ± 0.7 vs. 6.0 ± 0.7, P = 0.003). So was the rs2707466-C allele. The rs2908004-GG genotype had smaller right cuneus than rs2908004-GA (2.7 ± 0.4 vs. 2.8 ± 0.5, P = 0.002). So was the rs2707466-CC genotype relative to rs2707466-CT. The rs3762719-T allele was relevant to smaller right bank of superior temporal sulcus (TT, TC vs. CC: 1.4 ± 0.4, 1.4 ± 0.3 vs. 1.7 ± 0.3, P = 0.002). Also, the rs12639607-GA exhibited smaller right bank of superior temporal sulcus than rs12639607-AA (1.4 ± 0.3 vs. 1.7 ± 0.3, P = 0.003). Besides, the rs4968281-TT genotype displayed smaller left superior frontal gyrus relative to rs4968281-TC (18.4 ± 2.2 vs. 20.1 ± 2.4, P = 0.002).

### Effect of *APOE*-ε4 on MRI Morphometry

**Fig. 3:**
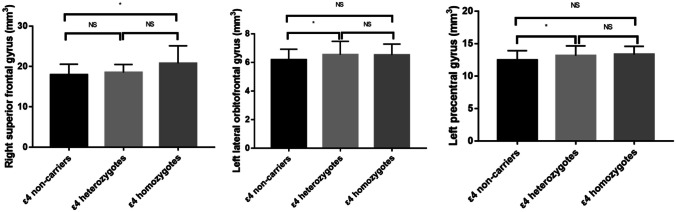
Effect of *APOE*-ε4 on frontal cortical volumes of AD patients. The MRI morphometry data were compared by analysis of covariate and post hoc Bonferroni correction. Age, gender, disease course and whole brain volume were included in the model as fixed factors or covariates. * P < 0.05; NS, P ≥ 0.05

As illustrated in Fig. 3, the *APOE*-ε4 was associated with greater frontal cortical volumes. The raw values were shown below (mm^3^). Compared with the *APOE*-ε4 non-carriers, the ε4 heterozygotes showed greater left lateral orbitofrontal gyrus (6.5 ± 0.9 vs. 6.2 ± 0.7, P = 0.014) and left precentral gyrus (13.1 ± 1.5 vs. 12.4 ± 1.5, P = 0.041). The ε4 homozygotes exhibited greater right superior frontal gyrus than ε4 non-carriers (20.8 ± 4.3 vs. 17.9 ± 2.6, P = 0.014).

The post hoc analysis with LSD correction demonstrated the smaller right hippocampus in ε4 homozygotes relative to ε4 non-carriers (3.0 ± 0.7 vs. 3.5 ± 0.7, P = 0.030). However, no statistical significance was found after Bonferroni correction.

## Discussion

This report focuses on the common variants of 32 Wnt target genes. Of them, the *APC*, *AXIN*, *DVL*, *FZD*, *LRP*, *TCF/LEF* and *WNT* families encode the vital elements of the Wnt signaling pathway. During the pathway, the WNT binds to LRP5/6 and FZD, which activates DVL and inhibits GSK-3β, AXIN, APC. Subsequently, the phosphorylation of ß-catenin is blocked, which contributes to the formation of TCF/LEF complex, and then the activation of the pathway (Jia et al. 2019; Li et al. 2005; Tapia-Rojas et al. 2016). The *DAAM1*, *DACT1*, *DISC1*, *LATS2*, *TLR2* and *WDR61* encode the regulators of the pathway (www.uniprot.org). The DAAM1 mediates DVL complex formation (Liu et al. 2008). The DACT1 interacts with DVL and regulates the degradation of β-catenin (Cheyette et al. 2002), (Zhang et al. 2006). The DISC1 modulates GSK-3β activity and CTNNB1 abundance (Mao et al. 2009). The LATS2 inhibits the β-catenin/BCL9 interaction (Li et al. 2013). The TLR2 participates in the WNT5A expression (Blumenthal et al. 2006). The WDR61 is the component of the PAF1 complex, which is required for the transcription of Wnt target genes (www.uniprot.org).

With the data from 51,665 individuals, Grasby et al., revealed that the loci affecting the cortical structures clustered near the genes related to the Wnt signaling pathway (Grasby et al. 2020). It is the first time that we describes the association between the Wnt target genes and cortical volumes in AD patients. In this study, 45.2% (38/84) Wnt target SNPs show effects on the cortical thickness of AD patients. We believe that the Wnt target genes affect the cortical thickness in both general population and AD patients.

Moreover, the Wnt target SNPs exert asymmetric effects on bilateral cortices of AD patients. The right temporal/parietal/occipital cortices are more affected than left temporal/parietal/occipital cortices. Nevertheless, the reverse applies to the frontal cortex. It is possibly owing to the asymmetric expression of Wnt target genes in the cortex. Miao et al., discovered that the predicted targets associated with the Wnt signaling pathway exhibited differential expression between the two hemispheres, such as the *WNT*, *FZD* and *AXIN2* (Miao et al. 2020). With a thorough review, Hüsken et al., concluded that the Wnt signaling pathway played a vital role in the establishment of brain asymmetry and laterality (Husken and Carl 2013).

In this study, the *DACT1* affects the cortical thickness most, followed by the *TCF3* and *APC*. The two SNPs with the most effects on the cortical volumes are rs698025 (*DACT1*) and rs863091 (*DACT1*). Grasby et al., also observed that the loci near the *DACT1* affected the cortical volume (Grasby et al. 2020). These findings emphasize the correlation between the *DACT1* gene and cortical thickness. Therefore, we advise to focus on the *DACT1* gene while exploring the genetic basis of cortical atrophy in AD patients.

The *APC* was identified as a novel AD susceptibility gene from a recent GWAS study (Prokopenko et al. 2021). However, no studies elucidated the relevance of the *APC* gene for AD phenotype. Herein, we find the *APC* gene is closely related to right posterior cortex. Five *APC* SNPs correlate with right calcarine cortex, and one SNP is relevant to right superior parietal lobule.

The brain region most influenced by the Wnt target genes is the right calcarine cortex. Eight SNPs show effects on the right calcarine cortex. This is different from the *APOE-*ε4, which is mainly associated with smaller hippocampal volume (Liu et al. 2015). In this report, we additionally find that the *APOE-*ε4 is related to greater frontal volume. This is consistent with two previous studies, which implies the region-specific effects of *APOE-*ε4 on the cortical atrophy of AD (Cacciaglia et al. 2018; Ten et al. 2016). We suppose that the Wnt target genes and *APOE* affect brain cortices of AD patients with different patterns.

The AD patients in this study show lower alternate allele frequencies in three *TCF3* SNPs (rs1052692, rs1052696, rs2074888) relative to the East Asian population. This may be due to the ethnic differences. The allele frequencies of these SNPs in Chinese population and AD patients could be further investigated.

In conclusion, the common variants of the Wnt target genes exert asymmetric effects on the cortical volumes of AD patients. The Wnt signaling pathway may play a role in the disproportionate cortical atrophy of AD patients. The main limitation of this report is the small sample size. The future research could focus on the *DACT1*, *APC* and *TCF3* genes with an expanded sample size.

### Supplementary Information

Below is the link to the electronic supplementary material.

Supplementary file1 (DOCX 57 KB)

## Data Availability

The original contributions are included in the article, further dataset are available from the corresponding author on reasonable request.
