# Supplementary material for: Association Between Wnt Target Genes and Cortical Volumes in Alzheimer’s Disease
Source: J Mol Neurosci. 2023 Dec 23;73(11-12):1010–6. doi: 10.1007/s12031-023-02122-1 (PMC10754720; doi:10.1007/s12031-023-02122-1)
Supplement: Supplementary file 1 — Supplementary file1 (DOCX 57 KB) [file 12031_2023_2122_MOESM1_ESM.docx]

**Supplement Table 1: 431 overlapping differentially expressed genes from Venn diagram of GSE132903, GSE33000, GSE63061**

| *ABCA2* | *ATP5F1* | *CASP4* | *COPS5* | *DPAGT1* | *GATAD2A* | *KIAA0556* | *MRPL21* | *NGDN* | *PPM1M* | *RFESD* | *SIRT2* | *TBC1D2* | *TXNDC9* |
| --- | --- | --- | --- | --- | --- | --- | --- | --- | --- | --- | --- | --- | --- |
| *ABCA7* | *ATP5J* | *CBX7* | *COX6C* | *DPM1* | *GDPD3* | *KIF1B* | *MRPL3* | *NME1* | *PPP1R14B* | *RGL2* | *SLC11A1* | *TBC1D20* | *UBE2T* |
| *ACADVL* | *ATP5J2* | *CCDC34* | *COX7A2* | *DPY30* | *GGA3* | *KLF2* | *MRPL32* | *NPEPL1* | *PRCP* | *RHBDF2* | *SLC15A4* | *TBC1D2B* | *UBE2V2* |
| *ACAT2* | *ATP5L* | *CCDC53* | *COX7B* | *DRG1* | *GIMAP7* | *KLF6* | *MRPL35* | *NT5C2* | *PRDX1* | *RIT1* | *SLC16A3* | *TBCA* | *UBL5* |
| *ACTN4* | *ATP5O* | *CCM2* | *CREBBP* | *DTWD2* | *GLRX3* | *KRCC1* | *MRPL39* | *NUBPL* | *PREX1* | *RPA3* | *SLC26A6* | *TCEAL4* | *UGP2* |
| *ACTR10* | *ATP6AP1* | *CCND3* | *CRISPLD2* | *DYNC1H1* | *GMFG* | *LARP7* | *MRPL42* | *NXF1* | *PRKAG1* | *RPAP3* | *SLC26A8* | *TCEAL8* | *UNC93B1* |
| *ACVR2B* | *ATP6V1D* | *CCT2* | *CRYZL1* | *DYNLT3* | *GNL3* | *LATS2* | *MRPL50* | *OCIAD1* | *PRMT1* | *RPL11* | *SLC44A2* | *TCEB1* | *UQCRB* |
| *ADAR* | *ATP6V1E1* | *CD14* | *CSAD* | *EBNA1BP2* | *GRN* | *LCAT* | *MRPS21* | *OCIAD2* | *PRPF18* | *RPL32* | *SNRPB2* | *TCIRG1* | *UQCRC2* |
| *AGPS* | *ATPIF1* | *CD68* | *CSF2RA* | *EEF1D* | *GTF2A2* | *LDHB* | *MRPS22* | *OPLAH* | *PSD4* | *RPL34* | *SNRPN* | *THOC3* | *UQCRH* |
| *AGTRAP* | *ATXN1* | *CD83* | *CSF3R* | *EEF1E1* | *GTF2B* | *LILRA2* | *MRPS28* | *OSBPL2* | *PSMA3* | *RPL4* | *SNURF* | *THOC7* | *UQCRQ* |
| *AIF1* | *BCCIP* | *CDAN1* | *CSNK1G2* | *EFHD2* | *GTF2H5* | *LILRB1* | *MTCH1* | *PAK1IP1* | *PSMA4* | *RPL41* | *SP1* | *THYN1* | *USP32* |
| *AIRE* | *BEX2* | *CDK5R1* | *CTAGE5* | *EIF3E* | *H2AFZ* | *LILRB3* | *MTIF2* | *PARP10* | *PSMA6* | *RPL6* | *SPAG7* | *TIMM23* | *VBP1* |
| *AK2* | *BEX4* | *CEBPB* | *CTNNA1* | *EIF3M* | *HIGD1A* | *LPP* | *MTMR3* | *PARP4* | *PSMB1* | *RPS14* | *SPEN* | *TLR2* | *VCAN* |
| *AKAP13* | *BICD2* | *CEBPD* | *CUX1* | *ELMO2* | *HINT1* | *LRCH4* | *MYADM* | *PDCD10* | *PSMB10* | *RPS17* | *SRP19* | *TMEM126A* | *VCL* |
| *AKAP7* | *BLZF1* | *CEBPZ* | *CWC15* | *EMG1* | *HLA-A* | *LSM14A* | *MYH9* | *PDCD2* | *PSMC2* | *RPS24* | *SRRM2* | *TMEM154* | *VRK1* |
| *AKR1C4* | *BNIPL* | *CETN2* | *DAPP1* | *ENY2* | *HMGB2* | *LSM3* | *MYL6* | *PDE4C* | *PSMC6* | *RPS6* | *SS18* | *TMEM175* | *WAS* |
| *ANAPC10* | *BOLA3* | *CETN3* | *DBI* | *ERH* | *HNMT* | *LSM5* | *MYO9B* | *PFDN1* | *PSMD10* | *RTN1* | *SSB* | *TMEM60* | *WDFY2* |
| *ANKRA2* | *BRPF1* | *CFDP1* | *DCLRE1C* | *ESD* | *HSP90AA1* | *LTB4R* | *N4BP2* | *PFDN5* | *PSMD14* | *RWDD1* | *SSBP1* | *TNS3* | *WDR61* |
| *ANKRD13D* | *BSDC1* | *CFLAR* | *DCTN1* | *EXOSC9* | *ICAM3* | *LYPLAL1* | *NBEAL2* | *PFKFB4* | *PSME1* | *S100A10* | *STAB1* | *TOMM20* | *YIF1B* |
| *ANKS1A* | *BTBD10* | *CHD2* | *DCTN6* | *FAM107B* | *ID3* | *MAP3K6* | *NDE1* | *PHF23* | *PTPRE* | *SAFB2* | *STAT1* | *TRAPPC4* | *ZBTB16* |
| *AOC2* | *C10orf54* | *CHMP5* | *DCUN1D5* | *FASTK* | *IGBP1* | *MAST3* | *NDUFA1* | *PILRA* | *PTRH2* | *SCAP* | *STAT2* | *TRERF1* | *ZC3H15* |
| *APH1B* | *C11orf1* | *CISD1* | *DDX1* | *FASTKD2* | *IGF2R* | *MCL1* | *NDUFA2* | *PKIA* | *PWP1* | *SCFD1* | *STK19* | *TRIM21* | *ZC3H3* |
| *APIP* | *C11orf58* | *CLCN7* | *DEF8* | *FCER2* | *IL10* | *MCM8* | *NDUFA4* | *PLCB2* | *RAB37* | *SCRIB* | *SUB1* | *TRIM56* | *ZCCHC17* |
| *ARHGAP4* | *C14orf2* | *CLDN9* | *DENND4B* | *FES* | *IL18* | *MED31* | *NDUFA6* | *PLOD1* | *RAB40C* | *SEC11A* | *SWAP70* | *TSPAN14* | *ZNF319* |
| *ARHGAP9* | *C1orf162* | *CNNM3* | *DENR* | *FGD3* | *ILF2* | *MICAL1* | *NDUFB2* | *PNPT1* | *RAD51C* | *SEC11C* | *SYF2* | *TSPO* | *ZNF430* |
| *ARHGEF11* | *C1QBP* | *CNPY2* | *DGAT1* | *FGR* | *ING3* | *MKNK1* | *NDUFB3* | *POLR2A* | *RAN* | *SEC61A1* | *SYNGR1* | *TTC25* | *ZNF493* |
| *ARMCX6* | *C20orf24* | *COASY* | *DGKD* | *FHOD1* | *ISY1* | *MLKL* | *NDUFB6* | *POMP* | *RARS* | *SELPLG* | *TAF1C* | *TUBA1A* | *ZNF766* |
| *ARPC3* | *C7orf43* | *COMMD1* | *DHX16* | *FRG1* | *ITGA5* | *MMP25* | *NDUFS4* | *PPA1* | *RBM34* | *SEMA4B* | *TAP1* | *TUBA4A* | *ZNHIT3* |
| *ASNSD1* | *C8orf37* | *COMMD3* | *DISC1* | *FXR1* | *ITGAX* | *MRPL13* | *NDUFS5* | *PPA2* | *RBM6* | *SH2D1A* | *TAPBP* | *TXN* |  |
| *ATG16L2* | *CALM2* | *COMMD8* | *DNAJA2* | *GAPDH* | *ITGB2* | *MRPL15* | *NDUFV2* | *PPFIA1* | *RDH14* | *SHFM1* | *TATDN1* | *TXNDC12* |  |
| *ATP2A2* | *CARD8* | *COPS4* | *DNAJC7* | *GAS7* | *KCNH3* | *MRPL20* | *NFKBIA* | *PPHLN1* | *REC8* | *SIN3B* | *TAX1BP1* | *TXNDC17* |  |

**Supplement Table 2: 84 Wnt target SNPs and alternate allele frequencies from GnomAD database**

| Gene | SNP | Detail | AAF* |
| --- | --- | --- | --- |
| *APC* | rs2229992 | NM_000038:exon12:c.T1458C:p.Y486Y | 0.67 |
| *APC* | rs351771 | NM_000038:exon14:c.G1635A:p.A545A | 0.82 |
| *APC* | rs41115 | NM_000038:exon16:c.G4479A:p.T1493T | 0.82 |
| *APC* | rs42427 | NM_000038:exon16:c.G5034A:p.G1678G | 0.82 |
| *APC* | rs866006 | NM_000038:exon16:c.T5268G:p.S1756S | 0.82 |
| *APC* | rs459552 | NM_000038:exon16:c.T5465A:p.V1822D | 0.90 |
| *APC* | rs465899 | NM_000038:exon16:c.G5880A:p.P1960P | 0.82 |
| *AXIN1* | rs214252 | NM_003502:exon7:c.T1827C:p.A609A | 0.06 |
| *AXIN1* | rs214250 | NM_003502:exon6:c.G1284A:p.S428S | 0.05 |
| *AXIN1* | rs1805105 | NM_003502:exon2:c.T762C:p.D254D | 0.30 |
| *AXIN2* | rs35415678 | NM_004655:exon8:c.C2062T:p.L688L | 0.27 |
| *AXIN2* | rs1133683 | NM_004655:exon6:c.C1386T:p.P462P | 0.35 |
| *AXIN2* | rs9915936 | NM_004655:exon6:c.A1365G:p.P455P | 0.82 |
| *AXIN2* | rs2240307 | NM_004655:exon2:c.T432C:p.I144I | 0.17 |
| *AXIN2* | rs2240308 | NM_004655:exon2:c.C148T:p.P50S | 0.32 |
| *DAAM1* | rs8022614 | NM_014992:exon5:c.G558A:p.K186K | 0.56 |
| *DAAM1* | rs941884 | NM_014992:exon12:c.A1389G:p.Q463Q | 0.81 |
| *DACT1* | rs17832998 | NM_016651:exon4:c.C1391T:p.A464V | 0.25 |
| *DACT1* | rs2003021 | NM_016651:exon1:c.C268T:p.L90L | 0.37 |
| *DACT1* | rs698025 | NM_016651:exon4:c.G2089A:p.G697S | 0.13 |
| *DACT1* | rs863091 | NM_016651:exon4:c.C1134T:p.V378V | 0.13 |
| *DISC1* | rs3738401 | NM_001164538 exon2 c.G791A p.R264Q | 0.25 |
| *DISC1* | rs3738402 | NM_001164538 exon5 c.C1393T p.L465L | 0.21 |
| *DISC1* | rs2492367 | NM_001164538 exon6 c.C1407T p.I469I | 0.15 |
| *DISC1* | rs12133766 | NM_001164538 exon9 c.G1863A p.L621L | 0.03 |
| *DISC1* | rs821616 | NM_001164538 exon11 c.A2110T p.S704C | 0.12 |
| *DISC1* | rs11122391 | NM_001164538 exon11 c.A2383G p.T795A | 0.03 |
| *DISC1* | rs821617 | NM_001164538 exon11 c.A2399G p.K800R | 0.12 |
| *DISC1* | rs2273890 | NM_001164538 exon7 c.1689+6T>C | 0.12 |
| *DVL2* | rs35594616 | NM_004422:exon14:c.A1662G:p.Q554Q | 0.49 |
| *DVL2* | rs222837 | NM_004422:exon8:c.G855A:p.Q285Q | 0.65 |
| *DVL2* | rs222836 | NM_004422:exon5:c.C621T:p.S207S | 0.48 |
| *DVL2* | rs2074216 | NM_004422:exon3:c.C405T:p.S135S | 0.38 |
| *FZD1* | rs139480179 | NM_003505:exon1:c.264_265insCCG:p.Q88delinsQP | 0.30 |
| *FZD10* | rs10848026 | NM_007197:exon1:c.G222C:p.L74L | 0.46 |
| *FZD3* | rs2241802 | NM_017412:exon5:c.A435G:p.L145L | 0.59 |
| *FZD6* | rs3736047 | NM_003506:exon2:c.A6G:p.E2E | 0.63 |
| *FZD6* | rs3808554 | NM_003506:exon4:c.A762G:p.L254L | 0.63 |
| *FZD6* | rs3808553 | NM_003506:exon4:c.A1033C:p.M345L | 0.63 |
| *FZD6* | rs1053917 | NM_003506:exon7:c.G2070A:p.P690P | 0.61 |
| *FZD8* | rs74989785 | NM_031866:exon1:c.C1227T:p.A409A | 0.13 |
| *LATS2* | rs59928188 | NM_014572 exon4 c.G1548A p.P516P | 0.07 |
| *LATS2* | rs558614 | NM_014572 exon4 c.C971T p.A324V | 0.53 |
| *LATS2* | rs7317471 | NM_014572 exon2 c.A81G p.K27K | 0.89 |
| *LATS2* | rs77919685 | NM_014572 exon4 c.C608T p.A203V | 0.04 |
| *LEF1* | rs4956157 | NM_016269:exon8:c.1008+10C>T | 0.97 |
| *LRP5* | rs314776 | NM_002335:exon5:c.884-4T>C | 0.11 |
| *LRP5* | rs4988319 | NM_002335:exon6:c.1412+8G>A | 0.11 |
| *LRP5* | rs545382 | NM_002335:exon8:c.T1647C:p.F549F | 0.99 |
| *LRP5* | rs2277268 | NM_002335:exon9:c.G1932A:p.E644E | 0.04 |
| *LRP5* | rs2306862 | NM_002335:exon10:c.C2220T:p.N740N | 0.20 |
| *LRP5* | rs4988322 | NM_002335:exon10:c.2318+6T>C | 0.04 |
| *LRP5* | rs556442 | NM_002335:exon15:c.G3357A:p.V1119V | 0.74 |
| *LRP5* | rs3736228 | NM_002335:exon18:c.C3989T:p.A1330V | 0.20 |
| *LRP6* | rs2302685 | NM_002336:exon14:c.G3184A:p.V1062I | 0.93 |
| *TCF3* | rs55677929 | NM_003200:exon8:c.500-8T>C | 0.68 |
| *TCF3* | rs62130064 | NM_003200:exon19:c.1823-8C>T | 0.03 |
| *TCF3* | rs2074888 | NM_003200:exon17:c.C1475T:p.A492V | 0.41 |
| *TCF3* | rs1140828 | NM_003200:exon15:c.C1308T:p.G436G | 0.26 |
| *TCF3* | rs8140 | NM_003200:exon15:c.A1302G:p.S434S | 0.87 |
| *TCF3* | rs1052696 | NM_003200:exon15:c.C1293T:p.G431G | 0.23 |
| *TCF3* | rs1052692 | NM_003200:exon15:c.G1291A:p.G431S | 0.23 |
| *TCF3* | rs2240590 | NM_003200:exon10:c.C759T:p.S253S | 0.21 |
| *TCF4* | rs8766 | NM_001243226:exon20:c.A2247G:p.S749S | 0.45 |
| *TCF7* | rs30489 | NM_001134851:exon9:c.G766A:p.G256R | 0.39 |
| *TCF12* | rs35615435 | NM_003205:exon3:c.A135G:p.Q45Q, | 0.30 |
| *TCF7L2* | rs77961654 | NM_030756:exon14:c.C1429A:p.P477T | 0.25 |
| *TLR2* | rs3804099 | NM_003264 exon3 c.T597C p.N199N | 0.29 |
| *TLR2* | rs3804100 | NM_003264 exon3 c.T1350C p.S450S | 0.26 |
| *WDR61* | rs3832993 | NM_025234 exon9 c.657-8C>- | 0.11 |
| *WDR61* | rs2280364 | NM_025234 exon5 c.G171A p.Q57Q | 0.13 |
| *WNT10B* | rs1051886 | NM_003394:exon5:c.C1059T:p.H353H | 0.46 |
| *WNT11* | rs1533767 | NM_004626:exon3:c.C408T:p.P136P | 0.20 |
| *WNT16* | rs2908004 | NM_057168:exon2:c.G244A:p.G82R | 0.18 |
| *WNT16* | rs2707466 | NM_057168:exon4:c.C788T:p.T263I | 0.18 |
| *WNT2B* | rs910697 | NM_004185:exon6:c.A1113G:p.Q371Q | 0.65 |
| *WNT7A* | rs3762719 | NM_004625:exon3:c.T459C:p.S153S | 0.55 |
| *WNT7A* | rs12639607 | NM_004625:exon3:c.G315A:p.A105A | 0.55 |
| *WNT8A* | rs6596422 | NM_058244:exon6:c.G741A:p.A247A | 0.59 |
| *WNT8B* | rs3793771 | WNT8B:NM_003393:exon1:c.G32C:p.C11S | 0.13 |
| *WNT9A* | rs8192629 | WNT9A:NM_003395:exon2:c.G270A:p.A90A | 0.08 |
| *WNT9A* | rs3795768 | WNT9A:NM_003395:exon2:c.T153C:p.A51A | 0.61 |
| *WNT9B* | rs4968281 | NM_003396:exon2:c.T317C:p.M106T | 0.44 |
| *WNT9B* | rs34072914 | NM_003396:exon3:c.G399T:p.R133R | 0.04 |

SNP: single nucleotide polymorphism; AAF*, alternate allele frequency in East Asian population from the GnomAD database.

**Supplement Table 3: Effect of Wnt target SNPs on MRI morphometry (raw value)**

| Gene | SNP | Brain region | Cortical volume (mm^3^) | P |
| --- | --- | --- | --- | --- |
| *APC* | rs351771 | Right calcarine cortex | GG/GA/AA: 2.7±0.7/2.3±0.5/2.1±0.4 | 0.004, GG vs. GA: 0.707, **GG vs. AA: 0.045, GA vs. AA: 0.031** |
| *APC* | rs41115 | Right calcarine cortex | GG/GA/AA: 2.7±0.7/2.3±0.5/2.1±0.4 | 0.004, GG vs. GA: 0.707, **GG vs. AA: 0.045, GA vs. AA: 0.031** |
| *APC* | rs42427 | Right calcarine cortex | GG/GA/AA: 2.7±0.7/2.3±0.5/2.1±0.4 | 0.004, GG vs. GA: 0.707, **GG vs. AA: 0.045, GA vs. AA: 0.031** |
| *APC* | rs465899 | Right calcarine cortex | GG/GA/AA: 2.7±0.7/2.3±0.5/2.1±0.4 | 0.004, GG vs. GA: 0.707, **GG vs. AA: 0.045, GA vs. AA: 0.031** |
| *APC* | rs866006 | Right calcarine cortex | TT/TG/GG: 2.7±0.7/2.3±0.5/2.1±0.4 | 0.004, TT vs. TG: 0.707, **TT vs. GG: 0.045, TG vs. GG: 0.031** |
| *APC* | rs459552 | Right superior parietal lobule | TA/AA: 11.4±1.4/10.6±1.5 | 0.004, **TA vs. AA: 0.004** |
| *AXIN1* | rs214250 | Left precentral gyrus | GG/GA/AA: 12.8±1.5/12.4±1.3/12.2 | 0.009, **GG vs. GA: 0.014**, GG vs. AA: 1.000, GA vs.AA: 0.812 |
| *AXIN1* | rs214252 | Left precentral gyrus | TT/TC/CC: 12.8±1.5/12.4±1.3/12.2 | 0.009, **TT vs. TC: 0.014**, TT vs. CC: 1.000, TC vs. CC: 0.812 |
| *AXIN2* | rs2240307 | Right superior temporal gyrus | TT/TC/CC: 9.8±1.5/10.4±1.4/10.4±1.6 | 0.024, **TT vs. TC: 0.041**, TT vs. CC: 0.334, TC vs. CC: 1.000 |
| *AXIN2* | rs2240307 | Right orbital gyrus | TT/TC/CC: 2.4±0.4/2.6±0.4/2.7±0.4 | 0.001, **TT vs. TC: 0.017, TT vs. CC: 0.017**, TC vs. CC: 0.433 |
| *AXIN2* | rs9915936 | Right parahippocampus | AA/AG/GG: 2.7±0.5/3.4±0.7/3.4±0.6 | 0.001, **AA vs. AG: 0.005, AA vs. GG: 0.001**, AG vs. GG: 1.000 |
| *AXIN2* | rs9915936 | Right insular cortex | AA/AG/GG: 4.8±0.6/6.1±0.8/6.0±0.8 | <0.001, **AA vs. AG: <0.001, AA vs. GG: <0.001**, AG vs. GG: 1.000 |
| *AXIN2* | rs9915936 | Right superior temporal gyrus | AA/AG/GG: 8.1±2.4/10.1±1.4/10.2±1.3 | <0.001, **AA vs. AG: 0.028, AA vs. GG: 0.007**, AG vs. GG: 1.000 |
| *DACT1* | rs698025 | Left hippocampus | GG/GA/AA: 3.4±0.6/3.2±0.5/2.6±0.7 | 0.022, GG vs. GA: 0.567, **GG vs. AA: 0.033**, GA vs. AA: 0.200 |
| *DACT1* | rs698025 | Right hippocampus | GG/GA/AA: 3.4±0.7/3.2±0.6/2.5±0.3 | 0.007, GG vs. GA: 0.152, **GG vs. AA: 0.026**, GA vs. AA: 0.307 |
| *DACT1* | rs698025 | Right temporal pole | GG/GA/AA: 2.4±0.4/2.0±0.6/2.2±0.3 | 0.005, **GG vs. GA: 0.005**, GG vs. AA: 0.810, GA vs. AA: 1.000 |
| *DACT1* | rs698025 | Left medial orbito-frontal gyrus | GG/GA/AA: 5.2±0.6/5.0±0.6/5.5±0.3 | 0.001, **GG vs. GA: 0.001**, GG vs. AA: 1.000, GA vs. AA: 0.165 |
| *DACT1* | rs863091 | Left hippocampus | CC/CT/TT: 3.4±0.6/3.2±0.5/2.6±0.7 | 0.022, CC vs. CT: 0.567, **CC vs. TT: 0.033**, CT vs. TT: 0.200 |
| *DACT1* | rs863091 | Right hippocampus | CC/CT/TT: 3.4±0.7/3.2±0.6/2.5±0.3 | 0.007, CC vs. CT: 0.152, **CC vs. TT: 0.026**, CT vs. TT: 0.307 |
| *DACT1* | rs863091 | Right temporal pole | CC/CT/TT: 2.4±0.4/2.0±0.6/2.2±0.3 | 0.005, **CC vs. CT: 0.005**, CC vs. TT: 0.810, CT vs. TT: 1.000 |
| *DACT1* | rs863091 | Left medial orbito-frontal gyrus | CC/CT/TT: 5.2±0.6/5.0±0.6/5.5±0.3 | 0.001, **CC vs. CT: 0.001**, CC vs. TT: 1.000, CT vs. TT: 0.165 |
| *DACT1* | rs2003021 | Right supramarginal gyrus | CC/CT/TT: 9.0±1.2/8.8±1.2/8.1±1.1 | 0.024, CC vs. CT: 0.426, **CC vs. TT: 0.030**, CT vs. TT: 0.319 |
| *DACT1* | rs2003021 | Right calcarine cortex | CC/CT/TT: 2.3±0.5/2.1±0.5/2.1±0.3 | 0.023, **CC vs. CT: 0.020**, CC vs. TT: 0.918, CT vs. TT: 1.000 |
| *DISC1* | rs3738401 | Left middle temporal gyrus | GG/GA/AA: 9.4±1.5/9.3±1.5/7.1±1.9 | 0.005, GG vs. GA: 1.000, **GG vs. AA: 0.004**, **GA vs. AA: 0.016** |
| *DVL2* | rs2074216 | Right temporal pole | CC/CT/TT: 2.2±0.6/2.4±0.3/2.4±0.3 | 0.035, **CC vs. CT: 0.029**, CC vs. TT: 0.817, CT vs. TT: 1.000 |
| *DVL2* | rs2074216 | Left frontal pole | CC/CT/TT: 1.1±0.2/1.2±0.2/1.2±0.2 | 0.015, **CC vs. CT: 0.012**, CC vs. TT: 0.539, CT vs. TT: 1.000 |
| *LATS2* | rs59928188 | Left temporal pole | GG/GA: 2.4±0.3/2.7±0.4 | 0.005, **GG vs. GA: 0.005** |
| *LATS2* | rs7317471 | Left medial orbito-frontal gyrus | AG/GG: 5.4±0.6/5.1±0.6 | 0.005, **AG vs. GG: 0.005** |
| *LATS2* | rs77919685 | Right transverse temporal gyrus | CC/CT: 0.8±0.1/0.9±0.1 | 0.049, **CC vs.CT: 0.049** |
| *LATS2* | rs77919685 | Right lateral orbito-frontal gyrus | CC/CT: 6.4±0.9/6.7±0.7 | 0.029, **CC vs.CT: 0.029** |
| *LRP5* | rs2277268 | Left superior frontal gyrus | GG/GA: 19.0±2.4/20.4±2.9 | 0.005, **GG vs.GA: 0.005** |
| *LRP5* | rs4988322 | Left superior frontal gyrus | TT/TC: 19.0±2.4/20.4±2.9 | 0.005, **TT vs.TC: 0.005** |
| *LRP5* | rs3736228 | Right calcarine cortex | CC/CT/TT: 2.1±0.5/2.3±0.5/2.3±0.4 | 0.046, **CC vs. CT: 0.041**, CC vs. TT: 1.000, CT vs. TT: 1.000 |
| *LRP6* | rs2302685 | Left insular cortex | GG/GA/AA: 5.7±0.6/5.5±0.7/5.9±0.8 | 0.004, GG vs. GA: 1.000, GG vs. AA: 0.726, **GA vs. AA: 0.004** |
| *LRP6* | rs2302685 | Right inferior temporal gyrus | GG/GA/AA: 7.5±0.6/9.6±1.7/9.6±1.6 | 0.038, GG vs. GA: 0.180, **GG vs. AA: 0.041**, GA vs. AA: 1.000 |
| *TCF12* | rs35615435 | Left superior temporal gyrus | AA/AG/GG: 10.8±1.4/9.8±1.4/9.5±1.7 | 0.011, AA vs. AG: 0.069, **AA vs. GG: 0.034**, AG vs. GG: 0.878 |
| *TCF3* | rs2240590 | Left insular cortex | CC/CT/TT: 5.9±0.7/5.5±0.7/6.3±0.8 | 0.004, **CC vs. CT: 0.003**, CC vs. TT: 1.000, CT vs. TT: 0.289 |
| *TCF3* | rs1052692 | Right inferior parietal lobule | GG/GA: 11.3±1.9/11.7±1.9 | 0.029, **GG vs. GA: 0.029** |
| *TCF3* | rs1052696 | Left medial orbito-frontal gyrus | CC/CT: 5.1±0.6/5.4±0.6 | 0.013, **CC vs. CT: 0.013** |
| *TCF3* | rs1052696 | Left inferior parietal lobule | CC/CT: 10.3±1.6/10.9±1.4 | 0.011, **CC vs. CT: 0.011** |
| *TCF3* | rs1052696 | Right inferior parietal lobule | CC/CT: 11.3±1.9/11.9±1.8 | 0.010, **CC vs. CT: 0.010** |
| *TCF3* | rs62130064 | Right superior temporal gyrus | CC/CT: 10.1±1.4/9.2±2.2 | 0.031, **CC vs. CT: 0.031** |
| *TCF3* | rs62130064 | Right inferior temporal gyrus | CC/CT: 9.6±1.6/8.4±2.6 | 0.046, **CC vs. CT: 0.046** |
| *TCF3* | rs1140828 | Right inferior parietal lobule | CC/CT/TT: 9.7±1.6/9.1±1.7/11.5 | 0.018, **CC vs. CT: 0.032**, CC vs. TT: 0.445, CT vs. TT: 1.000 |
| *TCF7* | rs30489 | Left lingual gyrus | GG/GA/AA: 5.6±1.0/5.8±0.8/6.0±0.9 | 0.049, GG vs. GA: 0.395, **GG vs. AA: 0.046**, GA vs. AA: 0.582 |
| *WNT16* | rs2707466 | Right lateral orbito-frontal gyrus | CC/CT/TT: 6.5±0.9/6.3±0.7/6.0±0.7 | 0.003, CC vs. CT: 0.882, **CC vs. TT: 0.002**, **CT vs. TT: 0.019** |
| *WNT16* | rs2707466 | Right cuneus | CC/CT/TT: 2.7±0.4/2.8±0.5/2.8±0.5 | 0.003, **CC vs. CT: 0.002**, CC vs. TT: 1.000, CT vs. TT: 0.427 |
| *WNT16* | rs2908004 | Right lateral orbito-frontal gyrus | GG/GA/AA: 6.5±0.9/6.3±0.7/6.0±0.7 | 0.003, GG vs. GA: 0.882, **GG vs. AA: 0.002**, **GA vs. AA: 0.019** |
| *WNT16* | rs2908004 | Right cuneus | GG/GA/AA: 2.7±0.4/2.8±0.5/2.8±0.5 | 0.003, **GG vs. GA: 0.002**, GG vs. AA: 1.000, GA vs. AA: 0.427 |
| *WNT7A* | rs12639607 | Right bank of superior temporal sulcus | GG/GA/AA: 1.4±0.4/1.4±0.3/1.7±0.3 | 0.004, GG vs. GA: 1.000, GG vs. AA: 0.053, **GA vs. AA: 0.003** |
| *WNT7A* | rs12639607 | Left supramarginal gyrus | GG/GA/AA: 9.0±1.2/10.0±1.8/10.2±1.6 | 0.030, **GG vs. GA: 0.032**, GG vs. AA: 0.126, GA vs. AA: 1.000 |
| *WNT7A* | rs3762719 | Right middle temporal gyrus | TT/TC/CC: 9.4±1.6/9.8±1.3/10.6±1.6 | 0.035, TT vs. TC: 0.574, **TT vs. CC: 0.030**, TC vs. CC: 0.245 |
| *WNT7A* | rs3762719 | Right bank of superior temporal sulcus | TT/TC/CC: 1.4±0.4/1.4±0.3/1.7±0.3 | 0.002, TT vs. TC: 1.000, **TT vs. CC: 0.041**, **TC vs. CC: 0.001** |
| *WNT8A* | rs6596422 | Right calcarine cortex | GG/GA/AA: 2.1±0.4/2.1±0.4/2.3±0.5 | 0.020, GG vs. GA: 1.000, GG vs. AA: 0.751, **GA vs. AA: 0.017** |
| *WNT8B* | rs3793771 | Left fusiform gyrus | GG/GC/CC: 7.9±1.5/8.9±1.2/9.1±0.6 | 0.011, **GG vs. GC: 0.033**, GG vs. CC 0.199, GC vs. CC: 1.000 |
| *WNT8B* | rs3793771 | Right middle temporal gyrus | GG/GC/CC: 9.7±1.5/10.6±1.3/9.4±1.5 | 0.049, **GG vs. GC: 0.049**, GG vs. CC 1.000, GC vs. CC: 1.000 |
| *WNT9A* | rs8192629 | Right middle temporal gyrus | GG/GA/AA: 9.9±1.5/10.1±1.2/13.4 | 0.019, GG vs. GA: 0.999, **GG vs. AA: 0.021**, GA vs. AA: 0.069 |
| *WNT9A* | rs8192629 | Left supramarginal gyrus | GG/GA/AA: 9.8±1.6/10.2±1.8/13.5 | 0.039, GG vs. GA: 1.000, **GG vs. AA: 0.044**, GA vs. AA: 0.116 |
| *WNT9B* | rs4968281 | Left superior frontal gyrus | TT/TC/CC: 18.4±2.2/20.1±2.4/18.9±3.0 | 0.002, **TT vs. TC: 0.002**, TT vs. CC: 0.104, TC vs. CC: 1.000 |
| *WNT9B* | rs34072914 | Right supramarginal gyrus | GG/GT: 8.8±1.2/10.2±0.5 | 0.026, **GG vs. GT: 0.026** |

As for each SNP, the MRI morphometry data were compared by analysis of covariate and post hoc Bonferroni correction. Age, gender, disease course, APOE status and whole brain volume were included in the model as fixed factor or covariate.
